# Research on the influencing factors and pathways of user health information disclosure behavior on medical and health platforms: based on PLS-SEM, NCA, and fsQCA analysis

**DOI:** 10.3389/fpubh.2025.1728302

**Published:** 2026-01-09

**Authors:** Chenxuan Yu, Yuqi Bi, Juncheng Jia, Siyu Liu, Kun Lv

**Affiliations:** 1 Business School, Ningbo University, Ningbo, China; 2 Ningbo University, Université of d'Angers Joint Institute, Ningbo, China; 3 College of Computer Science and Technology, Nanjing University of Aeronautics & Astronautics, Nanjing, China; 4 Faculty of Architecture and Art, Ningbo Polytechnic University, Ningbo, China; 5 Merchants' Guild Economics and Cultural Intelligent Computing Laboratory, Ningbo University, Ningbo, China

**Keywords:** digital health platforms, fuzzy set qualitative comparative analysis, health information disclosure, necessity analysis, structural equation modeling

## Abstract

With the rapid development of medical and health platforms, user health information disclosure behavior has become increasingly critical to the sustainable operation and effective dissemination of health information online. Given this context, understanding the underlying influencing factors and behavioral pathways is of great significance for promoting health information exchange and enhancing platform engagement. To this end, this study integrates the Technology Acceptance Model (TAM), Theory of Planned Behavior (TPB), and Knowledge-Attitude-Practice (KAP) framework to construct a comprehensive model of health information disclosure behavior. Data were collected from 824 users through online questionnaires and analyzed using partial least squares structural equation modeling (PLS-SEM), complemented by fuzzy set qualitative comparative analysis (fsQCA) and necessity analysis (NCA). The results reveal that perceived ease of use and perceived usefulness significantly and positively influence users' attitudes and willingness to disclose, while perceived trustworthiness and perceived behavioral control further enhance these effects. Moreover, both disclosure willingness and perceived behavioral control directly drive actual disclosure behavior. Through systematic configuration analysis, five distinct pathways to high-level information disclosure behavior are identified. These findings provide new theoretical insights into the mechanisms of user disclosure intention and offer practical implications for the sustainable development of digital health platforms.

## 1 Introduction

Medical and health platforms are online platforms that provide information services such as online consultation, access, sharing, and exchange of health information for individuals concerned about health issues and disease information. Major domestic medical and health platforms include professional software and websites such as “Dingxiang Doctor,” “Good Doctor Online,” and “China Medical and Health Network.” Supported by policies like the Healthy China strategy and “Internet Plus Healthcare,” medical and health platforms have developed rapidly, making public consultation, interaction, and sharing of health information more convenient (1).

The sustained growth of these platforms relies on active user participation, with health information disclosure being a critical component. Health information disclosure willingness refers to the degree to which users proactively share personal health data during platform usage. Sharing health information on these platforms not only helps users gain more insights into their own health conditions but also leverages the positive externalities inherent in health information itself to promote its dissemination and the popularization of health knowledge (2). Consequently, user health information disclosure is emerging as a new channel for disseminating healthcare information and enabling seamless online doctor-patient interactions. A primary objective of healthcare platform development is to enhance user motivation for health information disclosure, thereby increasing platform visibility and benefiting a broader user base. Exploring the factors influencing user health information disclosure can help platforms implement targeted measures to encourage health information exchange, boost user engagement, and ensure the sustained, stable operation of healthcare platforms.

## 2 Literature review

### 2.1 Behavioral theories in explaining online health information disclosure

The rise of digital health platforms has transformed the landscape of public health, making user engagement—particularly the disclosure of personal health information (HID)—a critical factor for effective knowledge dissemination and platform sustainability (3). To understand the cognitive and psychological mechanisms driving this behavior, research has primarily relied on established behavioral intention theories, notably the Technology Acceptance Model (TAM) and the Theory of Planned Behavior (TPB). The TAM (4) posits that a user's acceptance of a technology is determined by their Perceived Usefulness (PU) and Perceived Ease of Use (PEOU), which influence attitude and behavioral intention. It remains the dominant framework for studying user adoption of mobile health apps, electronic health records, and other health information systems. Furthermore, the TPB (5) extends this viewpoint by introducing the role of Subjective Norm (SN) and Perceived Behavioral Control (PBC), arguing that intention is the immediate antecedent to actual behavior. The TPB has been widely utilized in examining health-related intentions, such as searching for health information or the willingness to share sensitive data (6). While these models offer robust predictive power for intention, they primarily focus on technological and general psychological factors. A significant limitation is their constrained ability to capture the unique, deep-rooted health knowledge and underlying cognitive foundation of the users themselves, a crucial dimension that is particularly relevant in the high-stakes context of health information sharing.

### 2.2 The role of the KAP framework and limitations of existing integration

Given the contextual specificity of health behavior, the Knowledge-Attitude-Practice (KAP) framework is essential for understanding the progression from fundamental awareness to action. Originating from public health, KAP conceptualizes a linear causal chain: individuals must first acquire Knowledge about a health issue, which informs their Attitude toward the related behavior, ultimately leading to the desired Practice or action. Researchers have recognized the limitations of using a single theory, leading to attempts to integrate technology adoption models with health-specific theories, such as combining TAM with the Health Belief Model (HBM) to study health-related internet use (7). This trend validates the necessity of a multidisciplinary approach. However, a noticeable void remains in the current literature: few studies systematically integrate the complete KAP chain as a cognitive antecedent into the TAM and TPB structures to predict the actual disclosure behavior. Most integrated models either focus on intentions or incorporate only fragmented health beliefs. Consequently, we lack a comprehensive theoretical framework that successfully bridges the gap between an individual's foundational health literacy (KAP) and their technology-driven behavioral intentions (TAM/TPB), especially within the context of fast-evolving, large-scale Chinese digital health platforms.

### 2.3 Methodological gap: from sufficiency to necessity and configurations

Beyond the theoretical integration deficit, a critical methodological gap exists in online behavior research. Traditional methods like Partial Least Squares Structural Equation Modeling (PLS-SEM) primarily test for sufficiency, asking: “What factors can lead to high disclosure behavior?” These methods rely on symmetric relationships and averages, explaining overall causal effect. While valuable for prediction, they inherently fail to address two crucial aspects of complex social phenomena. First, they cannot identify necessary conditions (NCA), which ask: “What factors must be present for the outcome (disclosure behavior) to occur?” A condition might be necessary even if its correlation with the outcome is weak (8). Second, traditional methods treat constructs as independent and are unable to reveal the equifinality of high disclosure behavior, meaning there can be multiple, equally effective combinations or configurations of factors that lead to the same high outcome. Fuzzy-Set Qualitative Comparative Analysis (fsQCA) is designed specifically to uncover these complex, asymmetric configurational paths (9). Current literature predominantly uses only one of these methods. The failure to combine these analytical perspectives means that researchers and platform managers only gain partial insight into what is sufficient for disclosure, without understanding what is necessary and what alternative pathways exist for different user segments.

### 2.4 Statement of knowledge gap and research contributions

In summary, the knowledge gap this study seeks to address is three-fold: (1) The absence of a systematic, comprehensive framework that merges the user's foundational health cognition (KAP) with their technological and behavioral intentions (TAM/TPB) to fully explain the transition toward actual health information disclosure behavior. (2) Contextual Gap: the lack of empirical validation for such an integrated model specifically within the unique context of Chinese digital health platforms, which feature distinct cultural factors and regulatory environments. (3) Methodological Gap: the reliance on sufficiency-based analysis (PLS-SEM) and the resulting inability to identify the necessary conditions (NCA) and the complex configurational pathways (fsQCA) that leads to high disclosure behavior.

To bridge these gaps, this study constructs and validates an integrated KAP-TAM-TPB framework. Crucially, we employ a multi-method analytical strategy, combining PLS-SEM (to test causal paths and sufficiency), Necessary Condition Analysis (NCA) (to identify preconditions), and Fuzzy-Set Qualitative Comparative Analysis (fsQCA) (to uncover complex configurational recipes). This rigorous approach provides a novel and holistic understanding of the antecedent factors and complex causality underlying user health information disclosure behavior.

## 3 Theoretical framework and research hypotheses

### 3.1 Theoretical foundation

#### 3.1.1 Technology acceptance model

The Technology Acceptance Model comprises two primary factors: perceived usefulness and perceived ease of use (4). Perceived usefulness represents users' perception of the benefits health information disclosure brings to themselves; perceived ease of use reflects community users' perception of the convenience level of health information disclosure. Simultaneously, the Technology Acceptance Model posits that users' behavioral intention is determined by perceived usefulness and perceived ease of use through specific combinations of influencing factors. Here, behavioral intention refers to the intention to disclose health information, denoting the intensity of users' subjective willingness to share health information. Furthermore, perceived trustworthiness plays a crucial role in facilitating information disclosure, as this behavior requires mutual understanding and trust between information sharers and recipients. perceived trustworthiness reduces the perceived risks and costs associated with individual information disclosure, making it a significant factor influencing user information disclosure in online environments. Therefore, this project employs the Technology Acceptance Model (TAM) to analyze the factors influencing health information disclosure behavior among users of online health communities.

#### 3.1.2 Theory of planned behavior

The Theory of Planned Behavior builds upon the Technology Acceptance Model by incorporating variables such as behavioral attitude and perceived behavioral control, thereby expanding the model's applicability to a more objective level (10). Behavioral attitude represents an individual's assessment of their preference for performing a specific behavior. In this project, behavioral attitude refers to the attitude toward health information disclosure, manifested both in users' willingness to post and share health information and in their interest in participating in related health information discussions. Perceived behavioral control refers to an individual's perception of the ease or difficulty of performing a specific behavior, reflecting their perception of factors that facilitate or hinder the behavior. Therefore, this project combines the Theory of Planned Behavior and the Technology Acceptance Model to ultimately identify six variables as influencing factors for health information disclosure behavior: perceived ease of use, perceived usefulness, perceived behavioral control, attitude toward health information disclosure, perceived trustworthiness, and willingness to disclose health information.

#### 3.1.3 Knowledge attitude practice

The Knowledge Attitudes Practice (KAP) theory posits that individuals follow a “knowledge-belief-practice” process when making behavioral decisions (11). Specifically, an individual's cognition and knowledge establish the foundation for behavior, forming the beliefs and attitudes that underpin behavioral decisions, which in turn influence the occurrence of behavior. Within this framework, knowledge refers to the behavioral information perceived and mastered by individuals, serving as the foundation for action. In this study, knowledge encompasses the information users perceive regarding online health information disclosure, including perceived ease of use, perceived usefulness, perceived behavioral control, and perceived trustworthiness. Beliefs, formed through the internalization of knowledge, represent subjective cognition and intention states, including value judgments about the behavior and implementation intentions, acting as the driving force for eventual action. In this project, attitudes toward health information disclosure and disclosure intentions align with the fundamental definition of belief. The “knowledge-belief-practice” theory's concept of behavior, within this study's framework, corresponds to health information disclosure behavior. Therefore, based on the Technology Acceptance Model and Theory of Planned Behavior, this project identifies the influencing factors of health information disclosure behavior. Guided by the “knowledge-belief-practice” theory, it clarifies the fundamental logic of interaction among these factors.

### 3.2 Research hypotheses and model construction

#### 3.2.1 Analysis of factors influencing attitudes toward health information disclosure

According to the fundamental principles of the Technology Acceptance Model, perceived ease of use and perceived usefulness significantly influence the formation of behavioral attitudes. Based on the definition of perceived ease of use, it is clear that it represents the cost an individual incurs when adopting a certain behavior, thereby determining their attitude toward that behavior to a certain extent.

This study posits that users' perceived ease of use of online health platforms manifests specifically as their belief that navigating various sections of the platform is convenient. This perceived convenience reduces the time and effort required for information disclosure, fostering positive perceptions toward sharing health information and cultivating favorable behavioral attitudes.

Simultaneously, perceived usefulness significantly influences behavioral attitudes. This study posits that when users perceive health information disclosure on the platform as yielding positive feedback for themselves and benefiting others, it fosters a favorable attitude toward health information disclosure behavior.

Furthermore, perceived trustworthiness has been demonstrated to influence the formation of behavioral attitudes. This study posits that perceived trustworthiness in health information disclosure primarily manifests as users' belief that online health platforms can deliver positive health benefits and that the disclosure process will not result in negative consequences such as personal information leaks. Consequently, perceived trustworthiness among healthcare platform users fosters positive attitudes toward disclosure behavior.

Therefore, the hypotheses are:

**H1:** Perceived ease of use has a significant positive effect on attitudes toward health information disclosure.

**H2:** Perceived usefulness significantly and positively influences attitudes toward health information disclosure.

**H3:** Perceived trustworthiness significantly and positively influences attitudes toward health information disclosure.

#### 3.2.2 Analysis of factors influencing willingness to disclose health information

The Theory of Planned Behavior and the Technology Acceptance Model suggest that both perceived usefulness and behavioral attitude influence behavioral intention. In this study, when platform users perceive health information disclosure as beneficial to both themselves and other users, it promotes their intention to disclose health information.

Concurrently, the Technology Acceptance Model posits that behavioral attitudes influence behavioral intention. This study suggests that the more positive users' attitudes toward health information disclosure are, the more they perceive such disclosure as yielding positive health benefits, thereby generating the intention to disclose health information.

Moreover, perceived trustworthiness exerts a positive influence on behavioral intention. This study defines perceived trustworthiness as: (1) Trust in the platform itself—users' recognition of the platform's information processing capabilities and privacy protection methods, believing it safeguards the authenticity of platform information and prevents leakage or misuse of user data; (2) Trust in other platform users—users' belief that commitments and statements made by other platform users are reliable. Thus, robust trust relationships positively impact users' willingness to disclose information.

Hypotheses:

**H4:** Perceived usefulness significantly and positively influences willingness to disclose health information.

**H5:** Attitude toward health information disclosure significantly and positively influences willingness to disclose health information.

**H6:** Perceived trustworthiness significantly and positively influences willingness to disclose health information.

#### 3.2.3 Analysis of factors influencing health information disclosure behavior

Health information disclosure behavior is the subject of this study. Guided by the Theory of Planned Behavior, Technology Acceptance Model, and the “Knowledge-Belief-Practice” theory, perceived ease of use, perceived usefulness, perceived trustworthiness, attitude toward health information disclosure, perceived behavioral control, and intention to disclose health information all exert direct or indirect influences on health information disclosure behavior. This research posits that perceived behavioral control and intention to disclose health information are the two variables with direct influence on health information disclosure behavior.

First, within the Theory of Planned Behavior, perceived behavioral control refers to an individual's perception of the ease or difficulty of performing a specific behavior. This study posits that users' perceived behavioral control should encompass both internal and external dimensions: the internal dimension refers to users' subjective capacity for health information disclosure, including their ability to collect, process, edit, and publish information; The external aspect refers to the objective conditions provided by the platform for users to disclose health information, including the platform's assistance in guiding users, processing information, pushing information, providing feedback, and protecting privacy. Perceived behavioral control represents the decision-maker's perceived ability to control and the perceived difficulty of implementing a specific behavior. When individuals perceive their behavior as easily controllable, they are more likely to engage in the behavior provided other conditions are met. Conversely, when individuals perceive they have limited time and resources to control the behavior, their behavioral practice is constrained.

Within the Theory of Planned Behavior, intention is the direct determinant of behavior; the stronger an individual's intention, the greater the likelihood of behavior implementation.

Therefore, we hypothesize:

**H7:** Perceived behavioral control significantly and positively influences health information disclosure behavior.

**H8:** Health information disclosure intention significantly and positively influences health information disclosure behavior.

**Figure 1:**
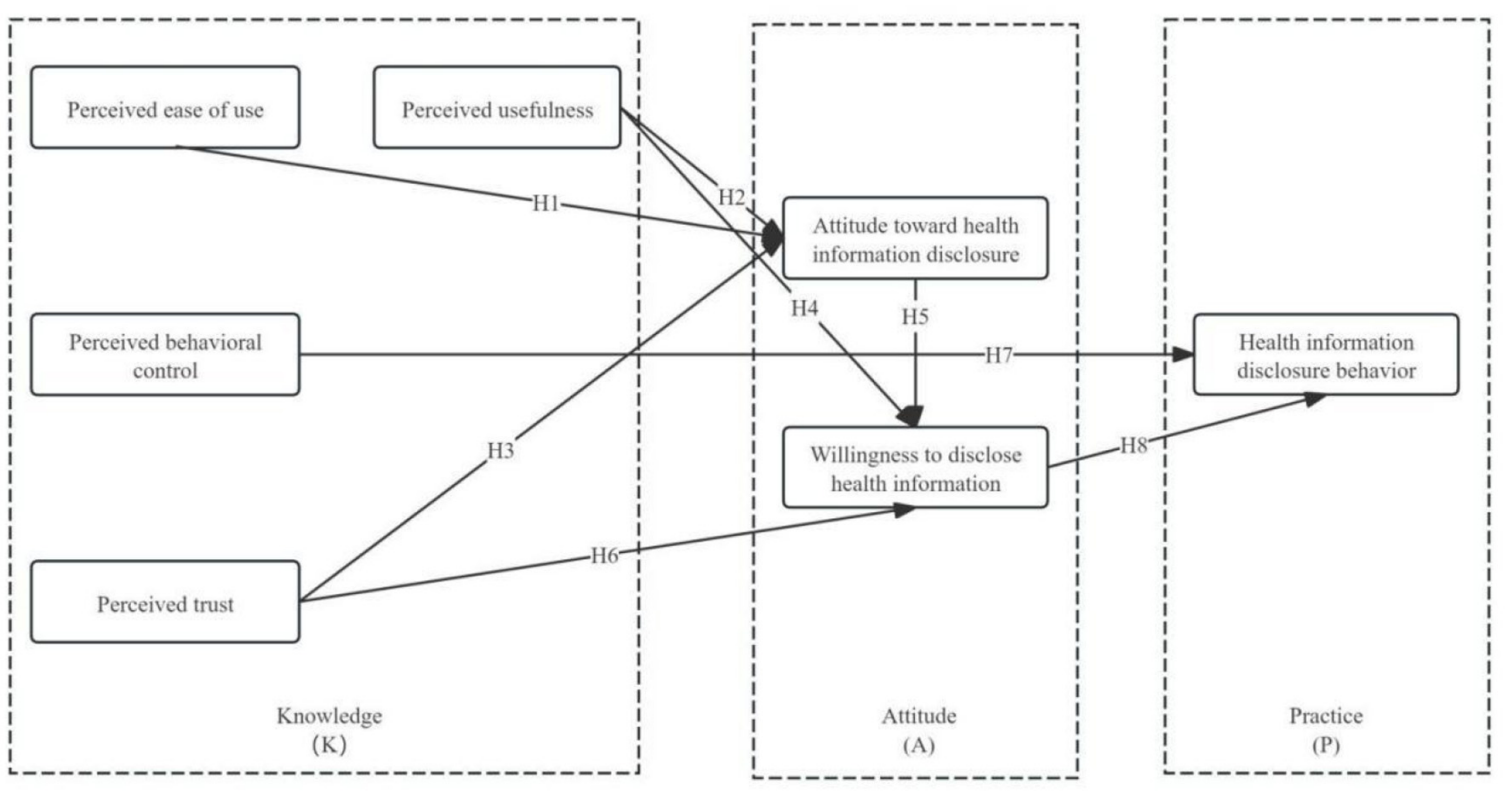
Theoretical framework of health information disclosure behavior among medical health platform users.

In summary, the model of health information disclosure behavior among medical health platform users is illustrated in Figure 1.

## 4 Data processing and structural equation modeling validation

### 4.1 Data source

We conducted a preliminary qualitative study to contextualize the theoretical framework derived from the literature. We recruited 15 active users (defined as users who post >3 times a week) from major Chinese health platforms, including “Dingxiang Doctor” and “Good Doctor Online.” Semi-structured interviews were conducted to verify the relevance of the proposed constructs (TAM, TPB, and KAP) to their actual usage experiences. The insights from approximately 40 h of interview recordings helped refine the measurement items tailored to the Chinese digital health context. A questionnaire survey of these platforms has been completed, yielding a total of 1,268 responses with 824 valid questionnaires.

As shown in Table 1, the questionnaire's observed variables were measured using a 5-point Likert scale. Each variable comprised a set of statements with response options ranging from “Strongly Agree” to “Strongly Disagree,” corresponding to scores of 5, 4, 3, 2, and 1, respectively. Additionally, after collecting the questionnaires, invalid responses that did not meet the survey objectives were excluded using the algorithm provided by Wenjuan Xing.

**Table 1:** Measurement table of questionnaire variables.

**No**.	**Variables**	**Measurement Item Content**	**Source**
1	Perceived ease of use	A1 Sharing health information on healthcare platforms comes easily to me	(22)
		A2 Healthcare platforms feature simple interfaces and are easy to use	
		A3 Health information shared on platforms is highly relevant to topics and easy to disclose	
2	Perceived usefulness	B1 Health information on platforms helps me address certain health issues	(4, 23)
		B2 Understanding health information on platforms benefits my health	
		B3 Health information on platforms broadens my related knowledge	
3	Perceived behavioral control	C1 Whether I share health information on platforms is entirely up to me	(6)
		C2 I am confident I can disclose health information on the platform	
		C3 I believe I can successfully disclose health information on the platform	
4	Attitude toward health information disclosure	D1 I enjoy disclosing health information on healthcare platforms	(24)
		D2 The experience of disclosing health information on the platform brings me pleasure	
		D3 I believe disclosing health information on healthcare platforms can help others	
5	Perceived trustworthiness	E1 I believe the platform is trustworthy and will not arbitrarily disclose my personal information	(25)
		E2 I believe platform users are trustworthy	
		E3 I believe health information shared among platform members is authentic and reliable	
6	Health information disclosure awareness	F1 I am willing to share health information through healthcare platforms	(26)
		F2 I will attempt to share health information with healthcare platform users	
		F3 I will share health information through healthcare platforms whenever possible	
7	Health information disclosure behavior	G1 I will share health information on the platform, such as treatment methods and experiences	(3)
		G2 I frequently spend time sharing health information on the platform	
		G3 When discussing relevant health issues with platform users, I engage in ongoing conversations with them	

#### 4.1.1 Demographic characteristics and representativeness

To address potential sampling bias, we compared the demographic profile of our respondents with the general profile of Chinese internet users reported by the China Internet Network Information Center (CNNIC). The sample comprised 437 males (53%) and 387 females (47%). The majority of respondents were aged between 18 and 35 years (68%) and held a bachelor's degree or higher (80%).

While our sample appears slightly younger and more educated than the general population statistics from CNNIC, this distribution is consistent with the typical user profile of digital health platforms in China, as younger and well-educated individuals are the primary adopters of “Internet + Healthcare” services (27). Therefore, the sample is considered representative of the target population for this study.

#### 4.1.2 Instrument development and measures

The measurement instrument was developed through a rigorous three-stage process to ensure construct validity and content validity.

Stage 1: Qualitative Exploration and Contextualization. Prior to finalizing the questionnaire, we conducted semi-structured interviews with active platform users, accumulating nearly 40 h of recording material. This qualitative phase allowed us to identify key themes relevant to the Chinese context and verify the applicability of the theoretical constructs.

Stage 2: Scale Adoption and Translation. The measurement items were adapted from established scales in existing literature (e.g., 10, 13) to fit the context of online health information disclosure. Since the original scales were in English, a standard back-translation procedure (12) was employed. A bilingual researcher first translated the English items into Chinese, and another researcher translated them back into English to ensure semantic equivalence.

Stage 3: Pilot Testing. A pilot study was conducted with 50 users to assess the clarity and readability of the items. Based on their feedback, minor modifications were made to the wording to avoid ambiguity. The final questionnaire used a 5-point Likert scale ranging from 1 (“Strongly Disagree”) to 5 (“Strongly Agree”).

#### 4.1.3 Non-response bias analysis

To assess potential non-response bias, we compared early respondents (the first 25%) with late respondents (the last 25%) under the assumption that late respondents are similar to non-respondents (13). Independent sample *t*-tests were conducted on key demographic variables (gender, age) and the mean scores of principal constructs. The results showed no statistically significant differences between the two groups (*p* > 0.05). Thus, non-response bias is not considered a serious threat to the validity of this study.

#### 4.1.4 Data analysis strategy

The data analysis was performed in a two-phase hybrid methodology to achieve a robust and comprehensive understanding of the factors influencing user health information disclosure behavior.

##### 4.1.4.1 Partial least squares structural equation modeling (PLS-SEM)

In the first phase, Partial Least Squares Structural Equation Modeling (PLS-SEM) was employed to test the research hypotheses and quantify the net causal effects among the constructs. PLS-SEM was chosen over covariance-based SEM for its suitability in: (1) handling complex research models with a large number of observed and latent variables; (2) supporting exploratory research and prediction; and (3) handling non-normal data distributions, which is common in questionnaire-based social science data (14). The analysis was conducted using the SmartPLS 4.0 software, following standard procedures for assessing the measurement model (reliability and validity) and the structural model (path coefficients and R-squared).

##### 4.1.4.2 Configurational and necessity analysis (fsQCA and NCA)

The second phase adopted a configurational approach using Fuzzy Set Qualitative Comparative Analysis (fsQCA) and Necessary Condition Analysis (NCA). This hybrid method was essential to overcome the inherent limitation of PLS-SEM, which assumes a net effect (i.e., finding the average influence of one variable while holding others constant). Complex sociological issues, like user behavior, often arise from the interplay and combined effects of multiple factors (equifinality and conjunctural causality), which a net effect analysis cannot fully capture.

1) Fuzzy Set Qualitative Comparative Analysis (fsQCA): This method emphasizes that complex outcomes result from specific configurations (or “causal recipes”) of influencing factors. Given the inherent ambiguity in users' health information disclosure behaviors and the subjective nature of the questionnaire data, fsQCA is an appropriate method to explore the various configuration paths that lead to high disclosure behavior. By setting the measurement range of variables between [0, 1] through the calibration process, fsQCA assigns precise values to conditional variables (9) and identifies conditions that are sufficient for the outcome to occur.
2) Necessary Condition Analysis (NCA): While fsQCA primarily focuses on sufficiency, NCA is employed to specifically identify and detect necessary but not sufficient conditions affecting outcome variables (8). Although fsQCA can also identify necessary conditions, NCA offers a significant advantage by quantitatively reflecting the degree of necessity and analyzing the required levels of a necessary condition (X) for different levels of the outcome (Y). Therefore, by employing this hybrid fsQCA and NCA methodology, this study not only identifies the sufficient paths but also rigorously examines the must-have conditions (necessary conditions) that influence information disclosure willingness and, if present, determines the required effort levels these conditions exert.

### 4.2 Validity and reliability testing

To validate the validity and reliability of the questionnaire data, this study examined the reliability and validity of the sample data. Reliability testing included internal consistency and composite reliability assessments, evaluated using Cronbach's Alpha and CR values, respectively. As shown in the calculation results of Table 2, the Cronbach's Alpha values for all variable items exceed 0.7, indicating that the scale possesses good reliability. Additionally, the CR values also exceed 0.7, signifying that the composite reliability test has been passed.

**Table 2:** Reliability and validity analysis results.

**No**.	**Latent variable**	**Item**	**Factor loadings**	**Cronbach's α**	**CR**	**AVE**
1	Perceived ease of use	A1	0.808	0.734	0.849	0.653
		A2	0.835			
		A3	0.780			
2	Perceived usefulness	B1	0.833	0.715	0.840	0.637
		B2	0.756			
		B3	0.804			
3	Perceived behavioral control	C1	0.799	0.727	0.846	0.647
		C2	0.805			
		C3	0.809			
4	Attitude toward health information disclosure	D1	0.797	0.721	0.843	0.642
		D2	0.801			
		D3	0.806			
5	Perceived trustworthiness	E1	0.797	0.715	0.827	0.614
		E2	0.785			
		E3	0.769			
6	Health information disclosure awareness	F1	0.801	0.723	0.832	0.623
		F2	0.763			
		F3	0.803			
7	Health information disclosure behavior	G1	0.796	0.715	0.831	0.621
		G2	0.782			
		G3	0.786			

The purpose of validity testing is to verify whether latent variables can be accurately measured by the observed variables. Typically, the factor loadings of the observed variables should exceed 0.70, and the Average Variance Explained (AVE) values should be greater than or equal to 0.50 (15). Higher factor loadings and AVE values for each observed variable demonstrate better convergent validity (16).

### 4.3 Assessment of the PLS-SEM structural model

The model's explanatory power was assessed using the coefficient of determination (*R*^2^). As shown in Table 3, the *R*^2^ values for Attitude Toward Health Information Disclosure, Willingness to Disclose, and Disclosure Behavior are 0.644, 0.608, and 0.614, respectively. According to Hair et al. (14) and Cohen (17), *R*^2^ values exceeding 0.50 indicate a moderate-to-strong explanatory power in behavioral research. These results demonstrate that the integrated model effectively explains the variance in users' health information disclosure behavior, indicating substantial predictive relevance.

**Table 3:** Model fit.

**Variable**	**R^2^**	**Adjusted R^2^**
Attitude toward health information disclosure	0.644	0.642
Willingness to disclose health information	0.608	0.606
Health information disclosure behavior	0.614	0.613

## 5 Necessity analysis

### 5.1 Overview of data analysis

To comprehensively examine the factors influencing health information disclosure behavior, this study employed a multi-analytic approach combining PLS-SEM, NCA, and fsQCA. The analysis proceeded in three stages: First, PLS-SEM was used to test the proposed hypotheses and assess the net effects of individual variables. Second, NCA was conducted to identify necessary conditions that must be present for the outcome to occur. Finally, fsQCA was employed to explore the sufficient configurational paths leading to high levels of health information disclosure behavior, addressing the causal complexity, and equifinality.

### 5.2 Structural model assessment and hypothesis testing

#### 5.2.1 Hypothesis test results

**Figure 2:**
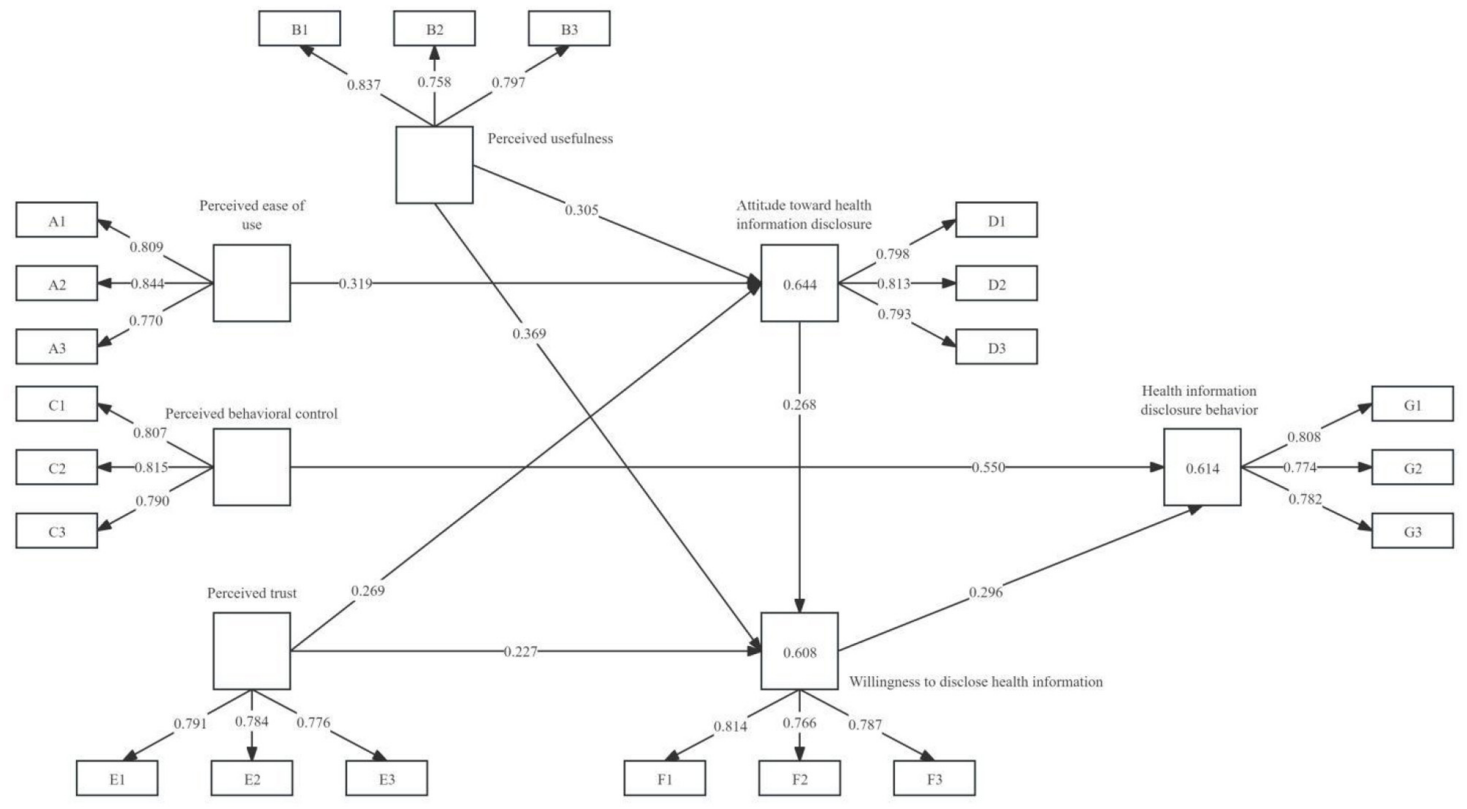
Results of the structural model for health information disclosure behavior.

The constructed model was tested using SmartPLS software, with results shown in Figure 2. Path coefficients were assessed for significance based on *t*-value magnitude to validate hypotheses, as presented in Table 4.

**Table 4:** Model hypothesis test results and path coefficients.

**Research hypothesis**	**Research pathway**	**Path coefficient**	***t-Value***	***p-Value***	**Hypothesis result**
H1	Perceived Ease of Use → Attitude Toward Health Information Disclosure	0.319	7.013	0.000	Support
H2	Perceived Usefulness → Attitude Toward Health Information Disclosure	0.305	6.906	0.000	Support
H3	Perceived Trustworthiness → Attitude Toward Health Information Disclosure	0.269	5.880	0.000	Support
H4	Perceived Usefulness → Willingness to Disclose Health Information	0.369	8.051	0.000	Support
H5	Attitude Toward Health Information Disclosure → Willingness to Disclose Health Information	0.268	5.908	0.000	Support
H6	Perceived Trustworthiness → Willingness to Disclose Health Information	0.227	5.243	0.000	Support
H7	Perceived Behavioral Control → Health Information Disclosure Behavior	0.550	13.929	0.000	Support
H8	Willingness to Disclose Health Information → Health Information Disclosure Behavior	0.296	7.066	0.000	Support

Table 4 reveals the following:

(1) H1 holds: Perceived ease of use exerts a significant positive influence on attitudes toward health information disclosure (path coefficient β = 0.319, *p-value* < 0.001). This indicates that individuals with higher perceived ease of use toward relevant matters are more inclined to adopt positive attitudes toward disclosing their health information, and this effect is neither coincidental nor isolated. For instance, when using simple and convenient online healthcare platforms, patients who find the interface easy to operate are more willing to provide their health status and medical history to the platform. This enables doctors to better understand their conditions, deliver accurate diagnoses and recommendations, and allows other platform users to access more information related to the disease.

(2) H2 holds: Perceived usefulness significantly and positively influences attitudes toward health information disclosure (path coefficient β = 0.305, *p-value* < 0.001). This indicates that in the healthcare domain, perceived usefulness fosters more positive attitudes toward health information disclosure by enabling users to access professional medical support, achieve self-management of health, promote efficient utilization of medical resources, and enhance health literacy.

(3) H3 holds: Perceived trust significantly and positively influences attitudes toward health information disclosure (path coefficient β = 0.269, *p-value* < 0.001). When users hold high trust in healthcare platforms themselves, medical professionals, and related institutions—believing their health information will be properly handled and protected without misuse or disclosure to unrelated third parties—they adopt a more positive stance toward disclosing health information.

(4) H4 holds: Perceived usefulness significantly and positively influences willingness to disclose health information (path coefficient β = 0.369, *p-value* < 0.001). This indicates that within specific contexts like healthcare platforms, users are more inclined to disclose their health information when they subjectively perceive the platform, tools, or services as highly practical for managing their health and accessing medical assistance.

(5) H5 holds: Attitude toward health information disclosure significantly and positively influences willingness to disclose health information (path coefficient β = 0.268, *p-value* < 0.001). Different users hold varying subjective evaluations and preferences regarding disclosing health information on healthcare platforms. The validity of this hypothesis indicates that users with a positive and optimistic attitude toward disclosing health information exhibit a stronger subjective intention to share personal health information on the platform than those with a pessimistic attitude.

(6) H6 holds: Perceived trust significantly and positively influences willingness to disclose health information (path coefficient β = 0.227, *p-value* < 0.001). Individuals exhibit greater openness to sharing their health data and information when they hold stronger trust in the entities collecting such information.

(7) H7 holds: Perceived behavioral control significantly and positively influences health information disclosure behavior (path coefficient β = 0.550, *p-value* < 0.001). This indicates that individuals' perceived behavioral control plays a crucial role in health information dissemination. When platform users believe they can effectively manage and share their health information, they are more likely to disclose it.

(8) H8 holds: Health information disclosure intention exerts a significant positive influence on health information disclosure behavior (path coefficient β = 0.296, *p-value* < 0.001). This implies that an individual's inclination to publicly share their health information—i.e., health information disclosure intention—significantly promotes the actual act of publicly disclosing health information.

#### 5.2.2 Model robustness testing

To ensure the robustness of the model fit, this study employed the Bootstrapping method for validation, with results presented in Table 5.

**Table 5:** Results of model robustness validation.

**Variable**	**Original sample**	**Sample mean**	**Standard error**	***T-value***	***P-value***	**Bias Value**	**2.50%**	**97.50%**
H1	0.319	0.321	0.046	7.013	0.000	0.002	0.230	0.409
H2	0.305	0.303	0.044	6.906	0.000	−0.001	0.220	0.393
H3	0.269	0.268	0.046	5.880	0.000	−0.001	0.182	0.359
H4	0.369	0.369	0.046	8.051	0.000	0.000	0.276	0.452
H5	0.268	0.267	0.045	5.908	0.000	−0.001	0.182	0.358
H6	0.227	0.227	0.043	5.243	0.000	−0.000	0.144	0.312
H7	0.550	0.551	0.040	13.929	0.000	0.000	0.469	0.624
H8	0.296	0.295	0.042	7.066	0.000	−0.000	0.215	0.380

If the Bias value of a parameter approaches zero, it indicates that the mean of the original sample estimate is very close to the mean of multiple self-sampled estimates. This suggests that the estimation results are relatively stable and less susceptible to sampling fluctuations. As shown in Table 5, the Bias values for all hypotheses in the health information disclosure behavior model are close to zero, indicating that the model performs consistently across different samples, i.e., it exhibits good stability. Additionally, Table 5 shows that the standard error of the sample mean for this model is small, further validating the reliability of the model's estimation results.

As shown in Table 6, the Q2 values for all variables exceed zero, indicating that the model proposed in this study exhibits strong predictive correlation and further confirming its robustness.

**Table 6:** Predictive correlation metric Q2.

**Variable**	**SSO**	**SSE**	**Q2 (= 1–SSE/SSO)**
Attitude toward health information disclosure	1,389.000	826.102	0.405
Willingness to disclose health information	1,389.000	871.948	0.372
Health information disclosure behavior	1,389.000	870.178	0.374

### 5.3 Necessary condition analysis

#### 5.3.1 Data calibration

Calibration refers to the process of adjusting antecedent conditions and outcomes to set membership scores between 0 and 1 based on anchor points. Prior to conducting conditional configural analysis on questionnaires, data must be calibrated to ensure antecedent and outcome variables possess set-theoretic meaning. Following Ragin's (18) methodology, this study calibrates data using 5%, 95%, and 50% intersection point thresholds. The 95th, 50th, and 5th percentiles of the sample serve as thresholds for complete membership, intersection points, and complete non-membership, respectively. Additionally, to prevent excessive cases at the 0.5 crosspoint from being omitted during calibration and affecting results, membership scores of 0.5 were manually adjusted to 0.501. Descriptive statistics for each variable are presented in Table 7, while calibrated anchor points are shown in Table 8.

**Table 7:** Descriptive statistics analysis results.

**Variable**	**Mean**	**Standard deviation**	**Minimum**	**Maximum**
Perceived ease of use	3.961123	0.8760064	1	5
Perceived usefulness	3.979122	0.8604564	1	5
Perceived behavioral control	3.945284	0.8841625	1	5
Attitude toward health information disclosure	3.949604	0.8789756	1	5
Perceived trustworthiness	3.897048	0.8623576	1	5
Willingness to disclose health information	3.923686	0.8694111	1	5
Health information disclosure behavior	3.966883	0.854052	1	5

**Table 8:** Variable calibrated anchor points.

**Variable**	**Full membership threshold**	**Crossover point**	**Full non-membership threshold**
Perceived ease of use	5.000	4.333	1.667
Perceived usefulness	4.667	4.333	1.667
Perceived behavioral control	5.000	4.333	1.667
Attitude toward health information disclosure	4.967	4.333	1.667
Perceived trustworthiness	5.000	4.000	1.700
Willingness to disclose health information	5.000	4.000	1.667
Health information disclosure behavior	4.667	4.333	1.667

#### 5.3.2 Necessary condition analysis

First, Necessary Condition Analysis (NCA) is employed to examine whether the outcome set constitutes a subset of a given condition set. Its primary metrics for determining necessary conditions are effect size and bottleneck level, where effect size represents the degree of constraint imposed by antecedent conditions on the outcome variable, and bottleneck level characterizes the maximum value antecedent conditions must attain when the outcome variable reaches its maximum value. NCA handles both continuous and discrete variables using upper bound regression (CR) and upper bound envelope (CE) methods, respectively. Table 9 presents the NCA-based analysis results. A condition is deemed necessary for the outcome when its effect size exceeds 0.1, the *p-value* test indicates statistical significance (*p* < 0.05), and the precision exceeds 95% (8). NCA analysis revealed that, except for the effect size of attitude toward health information disclosure being less than 0.1, the other five antecedent conditions met the requirements. Thus, perceived ease of use, perceived usefulness, perceived behavioral control, perceived trustworthiness, and awareness of health information disclosure are necessary conditions for the effective implementation of educational policies.

**Table 9:** Results of necessary condition analysis using NCA method.

**Condition**	**Method**	**Precision**	**Upper bound region**	**Range**	**Effect size d**	***P*-value**
Perceived ease of use	CR	99.6%	0.067	0.91	0.073	0.000
	CE	100%	0.094	0.91	0.104	0.000
Perceived usefulness	CR	99.6%	0.076	0.96	0.079	0.000
	CE	100%	0.134	0.96	0.139	0.000
Perceived behavioral control	CR	98.5%	0.089	0.91	0.097	0.000
	CE	100%	0.118	0.91	0.130	0.000
Attitude toward health information disclosure	CR	99.6%	0.049	0.92	0.053	0.010
	CE	100%	0.091	0.92	0.098	0.000
Perceived trustworthiness	CR	97.8%	0.103	0.91	0.113	0.000
	CE	100%	0.160	0.91	0.175	0.000
Willingness to disclose health information	CR	99.1%	0.082	0.91	0.090	0.000
	CE	100%	0.097	0.91	0.106	0.000

*d* < 0.1 indicates “low level”; the *p*-value represents the permutation test in NCA analysis (re-sampling count = 10,000). The closer the p-value is to 0, the more significant the effect.

Table 10 presents the bottleneck level analysis results for the NCA method. The table indicates that perceived trust emerges as the initial bottleneck factor for health information disclosure behavior. To achieve 100% disclosure, the following levels are required: 15.5% perceived ease of use, 16.5% perceived usefulness, 19.4% perceived behavioral control, 11.7% attitude toward health information disclosure, 22.4% perceived trust, and 19.4% awareness of health information disclosure.

**Table 10:** Bottleneck level analysis results.

**Health information disclosure behavior**	**Perceived ease of use**	**Perceived usefulness**	**Perceived behavioral control**	**Attitude toward health information disclosure**	**Perceived trustworthiness**	**Willingness to disclose health information**
0	NN	NN	0.0	NN	0.3	NN
10	0.7	1.1	2.0	0.0	2.5	0.6
20	2.4	2.8	3.9	1.3	4.7	2.7
30	4.0	4.5	5.9	2.6	6.9	4.8
40	5.7	6.2	7.8	3.9	9.1	6.9
50	7.3	7.9	9.7	5.2	11.3	9.0
60	8.9	9.6	11.7	6.5	13.6	11.1
70	10.6	11.4	13.6	7.8	15.8	13.2
80	12.2	13.1	15.5	9.1	18.0	15.3
90	13.9	14.8	17.4	10.4	20.2	17.3
100	15.5	16.5	19.4	11.7	22.4	19.4

This table was derived using the CR method.

NN, Not Necessary.

Building upon this, this study further employs the fsQCA method to validate the necessity of individual conditions for health information disclosure behavior. When the antecedent conditions leading to a specific outcome consistently exist and exhibit a consistency level exceeding 0.9, that condition is deemed a necessary condition for the outcome. As shown in Table 11, the consistency levels for individual antecedent conditions all fell below 0.9. This indicates that while each condition variable can partially explain the outcome variable, it is not a necessary condition for health information disclosure. Consequently, the level of health information disclosure behavior is influenced by multiple factors acting in concert, underscoring the necessity of conducting configurational analysis from a systems perspective.

**Table 11:** Necessity analysis of conditional variables.

**Condition**	**High-health information disclosure behavior**		**Non-high-health information disclosure behavior**	
	**Consistency**	**Coverage**	**Consistency**	**Coverage**
Perceived ease of use	0.760016	0.802605	0.642338	0.633227
~Perceived ease of use	0.652690	0.661577	0.799767	0.756751
Perceived usefulness	0.774486	0.758565	0.651603	0.595769
~Perceived usefulness	0.587285	0.643589	0.735941	0.752868
Perceived behavioral control	0.756375	0.809574	0.631669	0.631139
~Perceived behavioral control	0.655379	0.655893	0.809416	0.756187
Attitude toward health information disclosure	0.734158	0.778660	0.649966	0.643524
~Attitude toward health information disclosure	0.663897	0.670160	0.776446	0.731653
Trust	0.788045	0.725428	0.706679	0.607270
~Trust	0.573370	0.676793	0.680484	0.749816
Willingness to disclose health information	0.807180	0.729996	0.713949	0.602745
~Willingness to disclose health information	0.560741	0.677410	0.680183	0.767063

### 5.4 Configurational path analysis

#### 5.4.1 High-level health information disclosure configurational analysis

Constructing truth tables is a crucial step in conditional configurational analysis. Considering sample size and the number of antecedent conditions, this study sets the case frequency threshold to 1. Referencing priorresearch (19), the original consistency threshold is set to 0.8, while the PRI consistency is also set to 0.8 to reduce the likelihood of “concurrent subsets.”

Table 12 indicates that five configurational paths (H1, H2, H3, H4, H5) exist for high-level health information disclosure behavior. Each path exhibits individual consistency exceeding 0.9, with overall consistency also surpassing 0.9. Detailed analysis of the five configurational paths follows:

**Table 12:** Results of high-level health information disclosure behavioral configuration analysis.

**Conditions**	**H1**	**H2**	**H3**	**H4**	**H5**
Perceived ease of use	⊗			●	●
Perceived usefulness	●	●	●	●	
Perceived behavioral control		•		●	●
Attitude toward health information disclosure			•	•	•
Trust	•	•	•		•
Willingness to disclose health information	•	•	•	•	•
Original coverage	0.530	0.601	0.597	0.564	0.565
Unique coverage	0.009	0.010	0.008	0.020	0.022
Consistency	0.928	0.935	0.925	0.956	0.950
Overall consistency	0.905				
Overall coverage	0.682				

●indicates core condition present; ⊗indicates core condition absent; •indicates peripheral condition present; ⊗indicates peripheral condition absent; blank indicates condition optional, same applies below.

Configuration H1 exhibits a consistency of 0.928 and an original coverage of 0.53, explaining 53% of the sample cases. This configuration indicates that perceived usefulness serves as the core condition, while trust and willingness to disclose health information function as peripheral conditions. This suggests that even when perceived ease of use is low and perceived behavioral control and attitudes toward health information disclosure are unclear, combinations of other factors can still promote high-level health information disclosure behavior. This manifests as follows: even if a healthcare platform's operational convenience is low, users will spontaneously develop disclosure willingness and engage in disclosure behavior if they perceive health information disclosure as beneficial and trust the platform's protection of privacy data.

Configuration H2 has a consistency of 0.935 and an original coverage of 0.601, explaining 60.1% of the sample cases. This configuration indicates perceived usefulness as the core condition, with perceived behavioral control, trust, and willingness to disclose health information serving as peripheral conditions. Compared to H1, H2 adds perceived behavioral control as a peripheral condition. Individuals with clearer awareness of the ease of implementing disclosure behaviors can drive higher levels of disclosure, making H2 more explanatory than H1.

Configuration H3 exhibits a consistency of 0.925 and an original coverage of 0.597, explaining 59.7% of the sample cases. This configuration indicates that perceived usefulness serves as the core condition, while attitudes toward health information disclosure, trust, and willingness to disclose health information function as peripheral conditions. Building upon H1, holding an optimistic attitude toward disclosing health information further promotes high-level health information disclosure behavior.

Configuration H4 exhibits a consistency coefficient of 0.956 and an original coverage of 0.564, explaining 56.4% of the sample cases. This configuration indicates that perceived ease of use, perceived usefulness, and perceived behavioral control exist as core conditions, while attitude toward health information disclosure and willingness to disclose health information exist as peripheral conditions. This suggests that even in the absence of trust in the platform, combinations of other factors can still drive high levels of health information disclosure behavior. Furthermore, compared to other configurations, Configuration H4 exhibits the highest consistency, indicating it represents the optimal combination for fostering users' willingness to disclose information.

Configuration H5 exhibits a consistency of 0.950 and an original coverage of 0.565, explaining 56.5% of the sample cases. This configuration indicates that perceived ease of use and perceived behavioral control exist as core conditions, while attitude toward health information disclosure, trust, and willingness to disclose health information exist as marginal conditions. This suggests that even when perceived usefulness is lacking, if the platform is easy to operate, users possess strong disclosure capabilities, have high trust in the platform, and exhibit strong willingness to disclose information, it can still promote high-level information disclosure behavior.

#### 5.4.2 Non-high-level health information disclosure configural analysis

Causality in QCA lacks symmetry, so this paper further analyzes the “non-set” of outcome variables (i.e., non-high-level health information disclosure). One generated configuration path (NH1) can be regarded as an unfavorable factor influencing high-level health information disclosure behavior, as shown in Table 13.

**Table 13:** Results of non-high-level health information disclosure configural analysis.

**Conditions**	**NH1**
Perceived ease of use	⊗
Perceived usefulness	⊗
Perceived behavioral control	⊗
Attitude toward health information disclosure	⊗
Trust	⊗
Willingness to disclose health information	⊗
Original coverage	0.373
Unique coverage	0.373
Consistency	0.903
Overall consistency	0.903
Overall coverage	0.373

⊗indicates core condition absent; ⊗indicates peripheral condition absent.

In configuration NH1, willingness to disclose health information is absent as the core condition, while perceived ease of use, perceived usefulness, perceived behavioral control, attitude toward health information disclosure, and trust are absent as peripheral conditions. This configuration indicates that users lack the most fundamental willingness to disclose, and the platform's operational convenience and privacy protection capabilities are equally weak, making it difficult to achieve high-level health information disclosure behavior.

### 5.5 Robustness testing

Following the robustness testing method proposed by Yan and Junyao (20), we first increased the consistency index from 0.8 to 0.9 and observed that the configuration remained unchanged. Second, the PRI consistency threshold was raised from 0.8 to 0.85, yielding the configuration shown in Table 14. It can be observed that configuration S1 is fully consistent with H4, while a subset relationship exists between S2 and H5.

**Table 14:** Robustness test results 1.

**Conditions**	**S1**	**S2**
Perceived ease of use	●	●
Perceived usefulness	●	●
Perceived behavioral control	●	●
Attitude toward health information disclosure	•	
Trust		•
Willingness to disclose health information	•	•
Original coverage	0.564	0.567
Unique coverage	0.020	0.023
Consistency	0.956	0.950
Overall consistency	0.949	
Overall coverage	0.586	

●indicates core condition present; •indicates peripheral condition present.

Finally, by raising the case frequency threshold from 10 to 20, the resulting configurations are shown in Table 15. It can be observed that clear subset relationships exist between Configuration I1 and H1, as well as between I2 and H4. If the new configurations generated by different testing methods exhibit subset relationships with the original configurations, and the changes in consistency and coverage are insufficient to alter the substantive interpretation of the original configurations, this indicates highly robust result (21). In summary, the research results demonstrate good robustness.

**Table 15:** Robustness test results 2.

**Conditions**	**I1**	**I2**
Perceived ease of use	⊗	●
Perceived usefulness	●	●
Perceived behavioral control	●	
Attitude toward health information disclosure		●
Trust	•	●
Willingness to disclose health information	•	•
Original coverage	0.499	0.567
Unique coverage	0.047	0.115
Consistency	0.949	0.949
Overall consistency	0.933	
Overall coverage	0.614	

●indicates core condition present; •indicates peripheral condition present; ⊗indicates peripheral condition absent.

## 6 Discussion

### 6.1 General discussion

This study integrated TAM, TPB, and the KAP framework to construct a comprehensive model of health information disclosure behavior on medical platforms. The results from PLS-SEM largely supported our hypotheses. Consistent with the Technology Acceptance Model, both perceived ease of use and perceived usefulness were found to be significant predictors of users' attitudes and willingness to disclose (H1, H2, H4). This aligns with previous findings that functional benefits are primary drivers of user engagement in digital health contexts. Furthermore, the Theory of Planned Behavior constructs—perceived behavioral control and behavioral intention—were confirmed as direct determinants of actual disclosure behavior (H7, H8), reinforcing the importance of users' self-efficacy and subjective capabilities.

Uniquely, our findings highlight the critical role of trust. Perceived trustworthiness not only influences attitude but also directly impacts willingness to disclose (H3, H6). This suggests that in the virtual medical environment, where privacy risks are salient, trust acts as a fundamental prerequisite for information exchange.

### 6.2 Theoretical implications

Theoretically, this study makes two key contributions. First, by integrating KAP with TAM and TPB, we provide a more granular understanding of the “Knowledge-Belief-Practice” transformation process in digital health. While TAM and TPB focus on cognitive and intentional predictors, the KAP framework helps delineate the procedural progression from perception (Knowledge) to evaluation (Attitude/Belief) and finally to Action (Practice). Second, the application of fsQCA and NCA reveals the causal complexity of disclosure behavior. Unlike regression-based methods that assume symmetric relationships, our configurational analysis identified five distinct pathways (H1–H5) leading to high disclosure. For instance, we found that even when perceived usefulness is low, high trust combined with strong behavioral control can still drive disclosure (Configuration H5). This “equifinality” offers a theoretical explanation for why users with different motivations may exhibit similar engagement behaviors.

### 6.3 Practical implications

Based on our findings, platform managers should prioritize different strategies for different user segments. Since trust and perceived usefulness appear in multiple high-disclosure configurations, platforms must maintain strict privacy protection mechanisms while transparently communicating the utility of data sharing (e.g., better diagnosis accuracy). Additionally, given the significance of perceived behavioral control, simplifying the user interface to lower technical barriers is crucial for converting willingness into actual behavior.

## 7 Conclusions

### 7.1 Conclusions

This study explored the determinants of user health information disclosure behavior on medical platforms through a mixed-method approach. The PLS-SEM results confirmed that disclosure behavior is driven by a multi-dimensional mechanism involving utility (perceived usefulness), effort (ease of use), trust, and control capabilities. The fsQCA results further revealed that these factors do not act in isolation but combine in five distinct configurations to drive high-level disclosure. Specifically, we identified that while perceived usefulness is a core condition in most pathways, trust and behavioral control can act as compensatory mechanisms in specific contexts.

### 7.2 Limitations and future research directions

Despite the insights provided, this study has several limitations that suggest directions for future research. First, the cross-sectional data limits our ability to make strict causal inferences. Future research should employ longitudinal designs or experiments to track dynamic changes in user behavior over time. Second, our sample was limited to Chinese medical platforms, and the user base was relatively young and well-educated. Future studies should expand to other cultural contexts and include older demographics to verify the generalizability of the findings, particularly given the digital divide issues in older adult healthcare. Third, this study primarily focused on individual-level cognitive factors. Future research could incorporate external environmental variables, such as policy changes, platform incentive mechanisms, or social influence, to build a more holistic ecological model of health information disclosure.

## Data Availability

The original contributions presented in the study are included in the article/supplementary material, further inquiries can be directed to the corresponding author.
